# Guanxintai Exerts Protective Effects on Ischemic Cardiomyocytes by Mitigating Oxidative Stress

**DOI:** 10.1155/2017/4534387

**Published:** 2017-09-17

**Authors:** Jing Yang, Weiju Sun, Junfeng Sun, Fengyue Wang, Yuling Hou, Haiyan Guo, Hongyan Chen, Lu Fu

**Affiliations:** ^1^Department of Cardiovascular Medicine, The First Affiliated Hospital of Harbin Medical University, 23 Youzheng Street, Nangang District, Harbin 150001, China; ^2^Department of Coronary Care Unit, The First Hospital of Harbin, 151 Diduan Street, Daoli District, Harbin 150000, China; ^3^Department of Cardiovascular Medicine, The First People's Hospital of Luoyang, 88 East Zhongzhou Road, Luoyang 471000, China; ^4^Department of Cardiovascular Medicine, Heilongjiang Province Land Reclamation Headquarters General Hospital, 235 Hashuang Road, Nangang District, Harbin 150088, China

## Abstract

Oxidative stress participates in numerous myocardial pathophysiological processes and is considered a therapeutic target for myocardial ischemia and heart failure. Guanxintai (GXT), a traditional Chinese medicine, is commonly used to treat cardiovascular disease on account of its numerous beneficial physiological activities, such as dilating coronary arteries, inhibiting platelet aggregation, and reducing the serum lipid content. However, the antioxidative properties of GXT and potential underlying mechanisms remain to be established. In the present study, we investigated the protective effects of GXT on ischemic cardiomyocytes and the associated antioxidative mechanisms, both* in vivo* and* in vitro*. Notably, GXT treatment reduced the degree of cardiomyocyte injury, myocardial apoptosis, and fibrosis and partially improved cardiac function after myocardial infarction. Furthermore, GXT suppressed the level of ROS as well as expression of NADPH oxidase (NOX) and phospho-p38 mitogen-activated protein kinase (MAPK) proteins. Our results collectively suggest that the protective effects of GXT on ischemic cardiomyocytes are exerted through its antioxidative activity of NOX inhibition.

## 1. Introduction

Coronary heart disease (CHD) is a major cause of cardiovascular disease. In particular, acute myocardial infarction (AMI) and subsequent heart failure cause significant morbidity and mortality worldwide [1]. Myocardial infarction leads to activation of NADPH oxidase (NOX) and production of reactive oxygen species (ROS), in turn, activating mitogen-activated protein kinases (MAPKs), such as p38 MAPK, extracellular regulated protein kinases, and c-Jun N-terminal kinase [2]. Oxidative stress is involved in cardiomyocyte apoptosis, fibrosis, arrhythmia, and myocardial hypertrophy, which promote the development of heart failure after MI. Suppression of oxidative stress via knockdown of NOX2 and NOX4 is reported to attenuate cardiac remodeling and dysfunction [3], supporting the potential utility of antioxidative therapy as a novel strategy for MI.

Guanxintai (GXT), a traditional Chinese medicine commonly used to treat CHD, was approved by the State Food and Drug Administration of China in 1999. The treatment is developed according to pathogenesis of CHD and composed of Ginseng, Astragalus, Rehmannia, Ophiopogon root,* Schisandra chinensis*, Frankincense, Myrrh, Angelica root, Rhizoma Chuanxiong, Fructus liquidambaris, Radix cyathulae,* Salvia miltiorrhiza*, Acorusgramineus, and Motherwort. Clinical studies have confirmed beneficial effects of GXT on angina pectoris [4–7] and arrhythmia [8], in addition to inhibitory effects on lipid levels in blood [9]. A number of effective components of GXT, such as Astragalus and* Salvia miltiorrhiza*, have been shown to possess antioxidative properties [10, 11]. However, the precise mechanisms underlying the antioxidative activity of GXT remain to be elucidated. In the current study, we examined the protective effects of GXT on ischemic cardiomyocytes and the underlying antioxidative mechanisms, both* in vivo* and* in vitro*.

## 2. Materials and Methods

### 2.1. Animals and Ethics

Adult male Wistar rats (8 weeks old) with body weights of 220–250 g were purchased from the Laboratory Animal Center of the First Affiliated Hospital of Harbin Medical University of China. Animals were housed at a temperature of 22 ± 2°C and 50 ± 10% humidity under a 12 h light–dark cycle with free access to food and water. All experimental procedures were performed in accordance with the Guide for the Care and Use of Laboratory Animals of the National Research Council and approved by the Ethics Committee of the First Affiliated Hospital of Harbin Medical University.

Animals were randomly divided into three (sham-operated, MI, and GXT + MI) groups. The first two groups were intragastrically given normal saline (2.5 mL/kg/d) and the third group was given GXT (5 g/kg/d) for 3 days prior to operation. The MI model was generated by ligation of the left anterior descending coronary artery. After the rats were anesthetized with 10% chloral hydrate (3 ml/kg) by intraperitoneal injection, they were given mechanical positive pressure ventilation with a ventilator. The heart was quickly exposed and then the left anterior descending coronary artery was ligated with 3/8 needle and 6-0 sutures. Typical ST-segment elevation was used to confirm the success of the MI model. Sham-operated rats underwent an identical surgical procedure without coronary artery ligation, and all animals received normal saline or GXT (Shiyitang, Harbin, China) on the day of the operation lasting for 7 days. GXT was ground into powder and soaked in distilled water for 24 h and heated at 80°C to generate a final concentration of 2 g/mL [12, 13].

### 2.2. Echocardiography

Echocardiography was applied to assess cardiac structure and left ventricular function after GXT treatment using ultrasound apparatus (SONOS 7500; Philips, Rotterdam, The Netherlands) equipped with a 12 MHz transducer. Left ventricular end-diastolic dimension (LVEDD) and left ventricular end-systole dimension (LVESD) were measured, and left ventricular ejection fraction (LVEF) and fractional shortening (FS) were calculated from M-mode recordings. Measurements were analyzed by a blinded observer for three consecutive cardiac cycles. After completion of measurements, blood samples were collected from the heart and serum was obtained by centrifugation at 1000 ×g for 20 min. Then the hearts were rapidly removed, and part of the left ventricle was fixed in 4% paraformaldehyde or flash-frozen in liquid nitrogen until experimental use.

### 2.3. Enzyme Assays

The activities of serum creatine kinase-MB (CK-MB) and lactate dehydrogenase (LDH) were measured using detection kits (Nanjing Jiancheng Bioengineering Institute, Nanjing, China) according to the manufacturer's instructions.

### 2.4. Histopathological Analysis

After fixation in 4% paraformaldehyde, serial cross-sections (5 *μ*m) along the center of the fibrotic scar of left ventricles were obtained and stained with hematoxylin–eosin (HE) and Masson's trichrome to evaluate the changes in myocardial pathology and fibrosis. The fibrotic area percentage was determined by calculating the mean ratio of positive blue-stained connective tissue to total myocardium tissue area, which was used to determine the degree of cardiac fibrosis in the peri-infarct region. The terminal deoxynucleotidyl transferase mediated dUTP nick end labeling (TUNEL) assay was used to measure cells apoptosis following the instructions of the In Situ Cell Death Detection Kit, POD (Roche, Mannheim, Germany). Nuclei undergoing apoptosis were brown or brownish-yellow while normal nuclei were blue under a fluorescence microscope. Apoptotic index was calculated as the mean ratio of apoptotic nuclei to total nuclei of all measurements for each section. The percent of fibrosis area and apoptotic index were analyzed in 10 fields that were randomly selected from each section of the LV and were measured by using image analysis software (Image-Pro Plus 6.0, Media Cybernetics, Rockville, MD, USA).

### 2.5. Drug-Serum Preparation

Adult Wistar rats were grouped to GXT serum and blank serum groups and intragastrically given GXT (5 g/kg) or saline (2.5 mL/kg) orally every day for 7 days in succession, respectively. GXT was dissolved in distilled water at a final concentration of 2 g/mL. Following 1 h of treatment with drugs on day 7, blood was collected from rat abdominal aorta and centrifuged at 1000*g* for 20 min to acquire serum. After filtration through a 0.22 *μ*m cellulose acetate membrane, serum was bottled, heated to 56°C in water for 30 min, and stored at −20°C.

### 2.6. Cell Culture and Experimental Protocols

H9C2 cardiomyocytes (Cat 6110, Cell Biology, Shanghai, China) were purchased from the Shanghai Cell Biology Institute of China. Cells were cultured in high-glucose DMEM (Gibco, Carlsbad, California, USA) supplemented with 10% fetal bovine serum (Sciencell, Santiago, California, USA), 1% penicillin, and streptomycin (Beyotime, Shanghai, China) at 37°C under 5% CO_2_. Isoproterenol (ISO, Sigma Chemical Co., St. Louis, MO, USA) was used to establish the cardiomyocyte injury model. The ISO model group was treated with the NOX inhibitor, diphenyleneiodonium (DPI, Sigma Chemical Co.), or GXT serum. Blank serum-cultured H9C2 cardiomyocytes were taken as the control group.

### 2.7. Cell Viability Assay

H9C2 cardiomyocytes were seeded into 96-well plates and treated with 150 *μ*M ISO, 10 *μ*M DPI, GXT serum, or blank serum for 24 h. Cell viability was detected with the 3-(4,5-dimethylthiazol-2-yl)-2,5-diphenyltetrazolium bromide (MTT) assay (Amresco, Solon, USA). Since ISO and MTT interact with each other, the culture medium was replaced with 120 *μ*L MTT solution (5 mg/mL stock solution in PBS diluted with culture medium to a final concentration of 0.5 mg/mL). After 4 h incubation at 37°C, the solution was removed and the formazan produced solubilized in 150 *μ*L DMSO. Absorbance was measured using a microplate reader at 490 nm.

### 2.8. Flow Cytometry Analysis

Apoptosis was assessed with the Annexin V FITC/PI detection kit (Becton Dickinson, Franklin lakes, New Jersey, USA) according to the manufacturer's instructions. Briefly, H9C2 cardiomyocytes were seeded in six-well plates and incubated with 150 *μ*M ISO, 10 *μ*M DPI, culture solution of GXT serum, or blank serum for 24 h. Next, cells were collected and resuspended in 100 *μ*L binding buffer containing 5 *μ*L Annexin V FITC for 15 min at room temperature in the dark. After the addition of 300 *μ*L binding buffer containing 5 *μ*L PI, cells were subjected to flow cytometry.

### 2.9. Measurement of ROS

Intracellular ROS production in H9C2 cardiomyocytes and frozen rat heart tissues was analyzed using dihydroethidium (DHE) (GENMED, Shanghai, China). H9C2 cardiomyocytes were treated as described previously and frozen heart tissues incubated in the dark with 500 *μ*L DHE dissolved in Reagent C (500 : 1, GENMED) at 37°C in a CO_2_ incubator for 20 min. Fluorescence was detected under a fluorescence microscope with red light. The ROS content increased in proportion to the intensity of red fluorescence.

### 2.10. Western Blotting

Proteins of H9C2 cardiomyocytes and frozen heart tissues were extracted using RIPA buffer (Beyotime, Shanghai, China), followed by centrifugation at 12,000 ×g and 4°C for 10 min. Protein concentration was estimated with the Bicinchoninic Acid Protein Assay kit (Beyotime, Shanghai, China). Equal quantities of protein samples (60 *μ*g per lane) were separated via sodium dodecyl sulfate- (SDS-) polyacrylamide gel electrophoresis (PAGE) and transferred to polyvinylidene difluoride membrane (PVDF; Millipore, Bedford, MA, USA). After being blocked with 5% skimmed milk for 2 h at room temperature, the membranes were incubated with primary antibodies against NOX2 (1 : 1000, Abcam, Cambridge, MA, USA), NOX4 (1 : 500, Santa Cruz, Dallas, TX, USA), or GAPDH (1 : 5000, Kangchen, Shanghai, China) at 4°C overnight. Protein samples of H9C2 cardiomyocytes were additionally examined with primary antibodies against p38 MAPK (1 : 1000, CST, Beverly, MA, USA) and phospho-p38 MAPK (Thr180/Tyr182) (p-p38 MAPK; 1 : 1000, CST) at 4°C overnight. Then the membrane was washed with phosphate-buffered saline containing 0.5% Tween 20 three times for 10 min. Secondary goat antibody against rabbit or mouse IgG conjugated to horseradish peroxidase (1 : 2000, Zhongshan, Beijing, China) was added to membranes, depending on the source of primary antibodies, for 2 h. Membranes were subsequently visualized with enhanced chemiluminescence detection reagent (Thermo Scientific, Waltham, MA, USA) and exposed to X-ray film, which was digitized with a scanner (LiED 110; Canon, Tokyo, Japan). The band intensity (area × optical density) in each group was analyzed with Image J software (National Institutes of Health, Bethesda, MD, USA) and normalized to that of GAPDH, which was used as the internal control.

### 2.11. Real-Time PCR

Total RNA was extracted from frozen heart tissues with RNAiso Plus (Takara Bio, Otsu, Japan) and reverse transcribed to cDNA by using the PrimeScript RT Reagent kit with gDNA Eraser (Takara Bio) on a M-MLV Reverse Transcriptase (Takara, Dalian, China), according to the manufacturer's manual. Real-time PCR analysis was performed with SYBR Green (Roche, Mannheim, Germany) on an ABI 7500 RT-PCR system (Applied Biosystems, Foster City, CA, USA) to detect the mRNA level of* collagen (Col)I*, forward 5′-GACTGGCAACCTCAAGAAGG-3′, reverse 5′-GACTGTCTTGCCCCAAGTTC-3′ and* GAPDH, *forward 5′-GGAAAGCTGTGGCGTGAT-3′, reverse 5′-AAGGTGGAAGAATGGGAGTT-3′, which was used as an internal standard. The expression of* ColI* was quantified relative to GAPDH with the ΔΔCT method.

### 2.12. Statistical Analysis

All data are expressed as mean ± standard deviation. One-way analysis of variance (ANOVA), followed by Tukey post hoc test, was performed to determine differences among groups using SPSS 20.0 statistical software (SPSS Inc., Chicago, IL, USA). Data were considered statistically significant at *P* < 0.05.

## 3. Results

### 3.1. Effects of GXT on Cardiac Structure and Function after Myocardial Infarction

Echocardiogram results were recorded after 7 days of coronary artery ligation and GXT treatment (Table 1). Compared with the sham-operated group, we observed a significant increase in LVEDD and LVESD (all *P* < 0.01) and, conversely, a decrease in LVEF and FS (all *P* < 0.01) in the MI group, indicating heart dilatation and reduction of cardiac function. Administration of GXT alleviated this increase in LVEDD and LVESD (all *P* < 0.05), compared to the MI group. No significant differences were evident between the GXT + MI and MI groups in terms of LVEF and FS (all *P* > 0.05), although we observed a tendency of improvement after GXT treatment.

### 3.2. GXT Reduces Myocardial Histopathological Damage and Fibrosis after Myocardial Infarction

**Figure 1:**
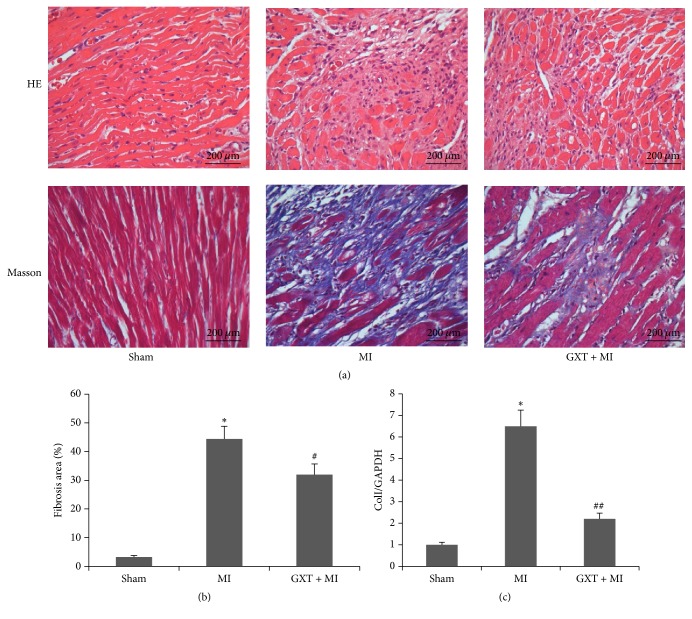
Effect of GXT on cardiac histopathology and myocardial infarct area. (a) Representative images (400× magnification) of HE and Masson's trichrome staining. (b) Quantitative analysis of fibrotic area. (c) mRNA expression level of* ColI *in the LV tissues. ^*∗*^*P* < 0.001 versus the sham group; ^#^*P* < 0.01 and ^##^*P* < 0.001 versus the MI group.

Within rats in the MI group, myocytes partially dissolved, degenerated, and necrosed, and muscular fibers were twisted, broken, and arranged in a disorderly manner, with inflammatory cell infiltration and interstitial hyperemia and edema, compared to those in the sham-operated group (Figure 1(a)). GXT treatment reduced the degree of damage to myocytes and interstitial cells as well as infiltration of inflammatory cells to a significant extent.

Masson's trichrome staining was applied to evaluate the degree of myocardial fibrosis (Figures 1(a) and 1(b)). Compared with the sham-operated group, the fibrosis area was significantly higher in the MI group (44.43 ± 4.40% versus 3.20 ± 0.53%, *P* < 0.001). The GXT treatment group displayed a significantly reduced degree of fibrosis (32.00 ± 3.66% versus 44.43 ± 4.40%, *P* < 0.01), compared with the MI group. As consistent with the degree of fibrosis, the mRNA level of* ColI* of MI rats was upregulated by approximately 5.5-fold, compared to sham rats (Figure 1(c), *P* < 0.001). GXT treatment inhibited the expression of* ColI* to 66% of the level in the MI group (*P* < 0.001).

### 3.3. GXT Alleviates Ischemic Cardiomyocyte Apoptosis

**Figure 2:**
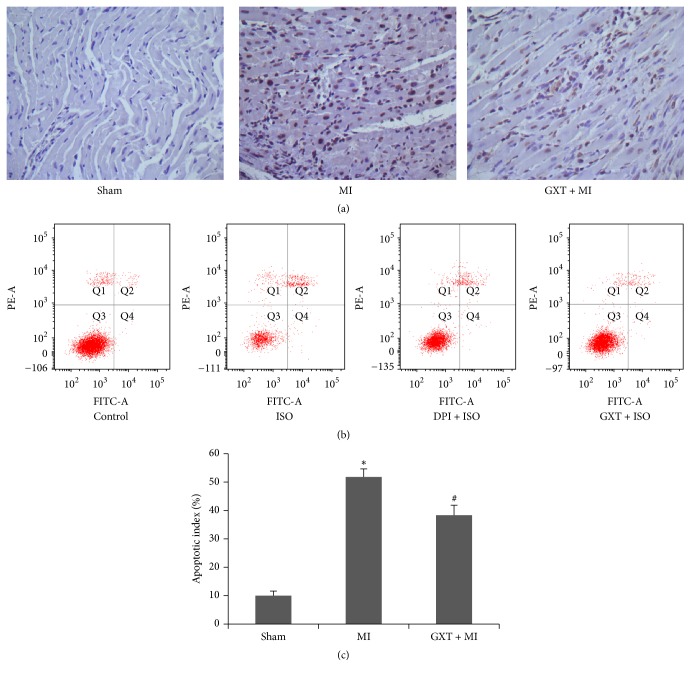
Effect of GXT on cells apoptosis. (a) Representative images (400× magnification) of TUNEL assay. (b) Flow cytometry analysis of apoptosis in H9C2 cardiomyocytes by using Annexin V FITC/PI double staining. (c) Quantitative analysis of apoptosis in the LV tissues. Values are mean ± SD from 3 rats (*n* = 3). ^*∗*^*P* < 0.001 versus the sham group; ^#^*P* < 0.01 versus the MI group.

The TUNEL method was used to detect cells apoptosis of myocardium (Figure 2(a)). The apoptotic index was significantly increased in the MI group (Figure 2(c), 51.80 ± 2.86% versus 10.00 ± 1.60, *P* < 0.001), compared to the sham-operated group. GXT treatment induced a significant decrease in the incidence of cell apoptosis (38.33 ± 3.51 versus 51.80 ± 2.86%, *P* < 0.01), compared with the MI group.

Apoptosis of H9C2 cells was determined with the Annexin V FITC/PI staining assay (Figure 2(b)). ISO triggered significant apoptosis, as determined from flow cytometry analysis, which was suppressed by GXT-containing serum or DPI.

### 3.4. Effect of GXT on the Serum Levels of CK-MB and LDH

**Figure 3:**
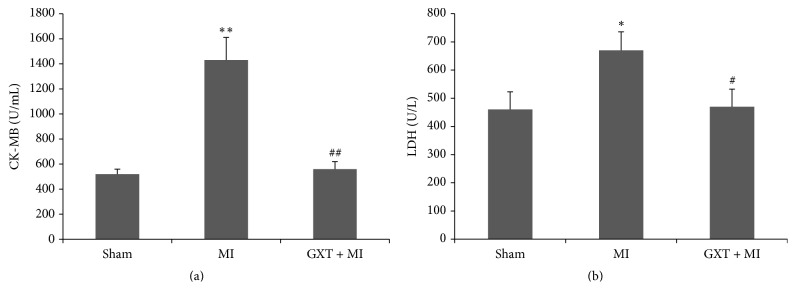
Effect of GXT on serum CK-MB (a) and serum LDH (b). Values are mean ± SD from 3 rats (*n* = 3). ^*∗*^*P* < 0.05 and ^*∗∗*^*P* < 0.001 versus the sham group; ^#^*P* < 0.05 and ^##^*P* < 0.001 versus the MI group.

CK-MB and LDH, reflecting cellular injury or tissue necrosis and membrane permeability, are regarded as diagnostic marker enzymes. The serum levels of CK-MB (Figure 3(a)) and LDH (Figure 3(b)) were significantly increased in the MI group (1430.00 ± 180.83 U/mL versus 520.00 ± 40.00 U/mL, *P* < 0.001; 670.00 ± 65.57 U/L versus 460.00 ± 62.45 U/L, *P* < 0.05, resp.), compared to the sham-operated group. GXT treatment significantly reduced the serum levels of CK-MB and LDH (560.00 ± 60.00 U/mL versus 1430.00 ± 180.83 U/mL, *P* < 0.001; 470.00 ± 62.45 U/L versus 670.00 ± 65.57 U/L, *P* < 0.05, resp.). These results indicated that GXT had protection on cardiomyocyte injury.

### 3.5. Effects of GXT on Cell Viability

**Figure 4:**
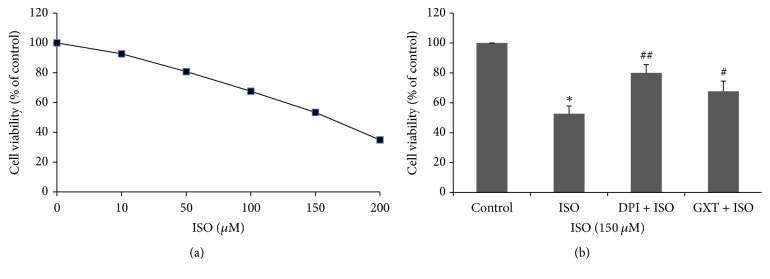
Cell viability determined by MTT assay. (a) Effects of different concentrations of ISO on cell viability. The preferred concentration of ISO was 150 *μ*M. (b) Effect of GXT on cell viability on ISO-treated H9C2 cardiomyocytes. Values are mean ± SD from 3 independent experiments. ^*∗*^*P* < 0.01 versus the control group; ^#^*P* < 0.05 and ^##^*P* < 0.01 versus the ISO group.

ISO (50–200 *μ*M) inhibited H9C2 cardiomyocyte survival after treatment for 24 h in a dose-dependent manner (Figure 4(a)). The concentration of ISO preferred for the cell line was 150 *μ*M. Following treatment, cell viability of the ISO group was decreased relative to that of the control group (Figure 4(b), 52.70 ± 5.18% versus 100.00 ± 0.00%, *P* < 0.01). GXT-containing serum and DPI ameliorated the reduction in cell viability caused by ISO (GXT: 67.73 ± 6.82%; DPI: 80.07 ± 5.50%, all *P* < 0.05).

### 3.6. GXT Suppresses ROS Production

**Figure 5:**
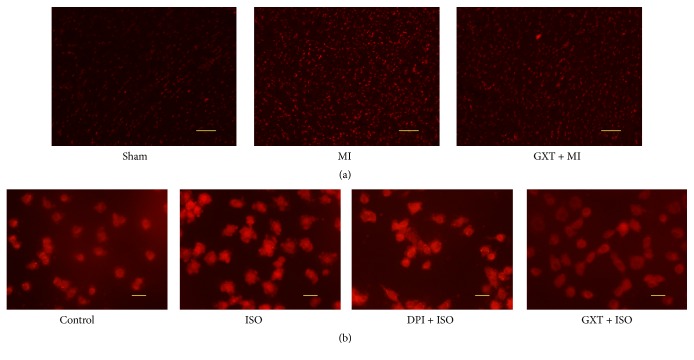
Effect of GXT on ROS levels in the LV tissues and H9C2 cardiomyocytes. (a) DHE staining in frozen sections by fluorescent microscopy (100× magnification). (b) DHE staining in H9C2 cardiomyocytes (400× magnification).

Frozen sections of myocardial tissues were prepared to detect ROS levels (Figure 5(a)). Compared with the sham-operated group, the ROS content was significantly increased in the MI group, indicating a higher concentration of red fluorescent particles. GXT treatment resulted in a decrease in the ROS level, compared to the MI group.

Analogously, ROS generation was significantly higher in ISO-treated cells relative to the control group in Figure 5(b). Increased production of ROS was markedly reduced in cells treated with GXT-containing serum or DPI, compared to the ISO group. Our results collectively suggest that stimulation of ROS by ISO is inhibited by GXT-containing serum.

### 3.7. GXT Inhibits NOX2 and NOX4 Expression

**Figure 6:**
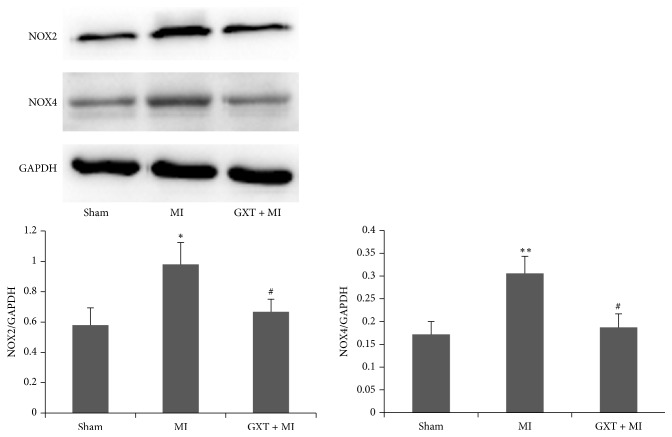
Effects of GXT on the protein expression of NOX2 and NOX4 in the LV tissues. The ratios of NOX2 and NOX4 to GADPH were calculated. Values are mean ± SD from 3 rats (*n* = 3). ^*∗*^*P* < 0.05 and ^*∗∗*^*P* < 0.01 versus the sham group; ^#^*P* < 0.05 versus the MI group.

Expression levels of NOX2 and NOX4 in the LV tissues of all the groups were analyzed via western blot (Figure 6). Compared with the sham-operated group, expression of NOX2 and NOX4 proteins was significantly increased in the MI group (all *P* < 0.05). Notably, GXT treatment led to marked suppression of NOX2 and NOX4 expression, compared to the MI group (all *P* < 0.05).

**Figure 7:**
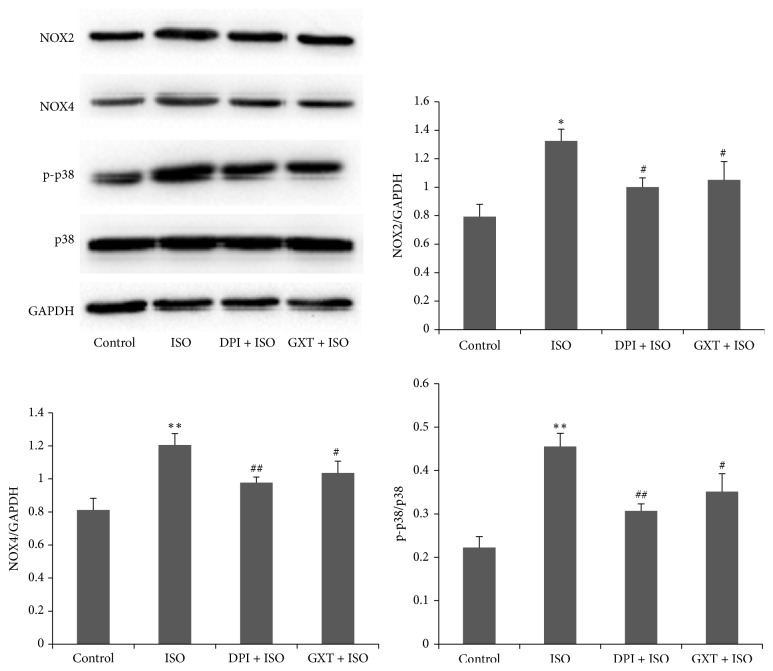
Effects of GXT on the protein expression of NOX2, NOX4, and p-p38 MAPK in H9C2 cardiomyocytes. The ratios of NOX2 and NOX4 to GADPH and the ratio of p-p38 MAPK to p38 MAPK were calculated. Values are mean ± SD from 3 independent experiments. ^*∗*^*P* < 0.01 and ^*∗∗*^*P* < 0.001 versus the control group; ^#^*P* < 0.05 and ^##^*P* < 0.01 versus the ISO group.

Consistently, NOX2 and NOX4 protein expression patterns in H9C2 cells exhibited a similar trend (Figure 7). ISO induced higher expression of NOX2 and NOX4, compared with the control group (*P* < 0.01). The levels of these proteins were lower in the GXT serum and DPI groups, compared to the ISO group (all *P* < 0.05). These findings indicate that the cell protective effects of GXT are exerted via its antioxidative stress activity.

### 3.8. GXT Inhibits p38 MAPK Activation

As shown in Figure 7, compared with the control group, phosphorylation of p38 MAPK was increased upon following treatment of cells with ISO (*P* < 0.001). GXT-containing serum or DPI suppressed the p-p38 MAPK/p38 MAPK ratio, compared to the ISO group (all *P* < 0.05).

## 4. Discussion

The GXT pill has been widely used for the treatment of CHD in clinics in China for decades. Several protective effects of GXT in patients with CHD have been documented, including coronary artery dilation, improvement of circulation, decrease in cardiac oxygen consumption, reduced area of MI, inhibition of platelet aggregation, and decreased serum lipid content [9, 14, 15]. In the current study, we evaluated the protective effect of GXT on ischemic cardiomyocytes in a rat model of MI and illustrated the potential mechanisms of antioxidative stress using a model of H9C2 cardiomyocytes cultured with GXT-containing serum* in vitro *according the serum pharmacological method [16].

Oxidative stress is increased in MI, and ROS appear to be involved in the processes of cardiomyocyte apoptosis, hypertrophy, interstitial fibrosis, and remodeling, which are closely associated with heart failure after MI. ROS could activate inflammatory cytokines that participate in cell death and apoptosis. ROS and inflammatory cytokines additionally activate interstitial metalloproteinase and collagenase, causing myofibril slippage, left ventricle dilation, and, consequently, collagen decomposition and myocardial fibrosis [17]. Therefore, oxidative stress is considered an effective therapeutic target for MI. Data from our study confirmed that oxidative stress aggravates myocardial damage after MI. In our experiments, the ROS content was significantly increased in the MI group, compared to the sham-operated group. We also observed the higher diagnostic marker enzymes levels, more serious myocardial pathological changes, and cardiac dysfunction, clearly supporting the involvement of oxidative stress in the process of left ventricle remodeling. In view of the improved myocardial pathological changes and smaller left ventricle as well as lower ROS levels in the GXT + MI group relative to the MI group, we propose that short-term intervention with GXT alleviates cardiomyocyte damage, apoptosis, and fibrosis by inhibiting oxidative stress after MI. Our results are in keeping with earlier findings by Dr. Zhang and colleagues that GXT shortens the duration of ischemia-reperfusion induced arrhythmia via antioxidative activities, such as reducing the myocardium malondialdehyde content and improving superoxide dismutase activity [18].

NOX proteins are the main source of ROS, which transfer one electron to O_2_ to generate O_2_^−^ and further ROS species. The heart primarily expresses NOX2 and NOX4 isoforms. Both experimental and clinical studies have consistently revealed upregulation of NOX2 after MI [19, 20]. Qin et al. showed that inhibition of NOX activity reduces oxidative stress and myocyte apoptosis and ameliorates LV dilation and dysfunction after MI [21]. Furthermore, NOX2 knockout mice display reduced post-MI remodeling and improved cardiac function [22, 23]. NOX4 is upregulated in the paraventricular nucleus after MI and its silencing shown to improve cardiac function by reducing sympathetic outflow from the brain and peri-infarct apoptosis in this setting [24]. The above studies collectively suggest that inhibition of NOXs reduces oxidative stress, in turn, ameliorating cardiac remodeling and dysfunction after MI. Data from the current investigation showed that NOX2 and NOX4 are upregulated and play an important role in increased production of ROS in the MI and ISO-induced cardiomyocyte injury models. Ischemia enhanced the ROS, NOX2, and NOX4 levels and aggravated myocyte apoptosis. GXT treatment induced a marked decrease in NOX2 and NOX4 expression, along with reduction of ROS and cardiomyocyte apoptosis. In view of these findings, we conclude that GXT alleviates oxidative stress during ischemia by inhibiting expression of NOX2 and NOX4.

To further investigate the mechanisms underlying the antioxidative activity of GXT, preliminary experiments were performed with the NOX inhibitor, DPI. In the GXT-containing serum group and DPI group, the intracellular ROS level decreased, cell viability increased, and NOX2, NOX4, and p-p38 MAPK protein levels were significantly downregulated, compared to the ISO group. p38 MAPK is an important intracellular signaling kinase involved in cell death regulation, which is activated by a range of stimuli, such as ROS [25]. Simultaneously, the apoptosis rate of H9C2 cells was significantly decreased, as determined from flow cytometry analysis, along with reduction of ROS and p-p38 MAPK expression.

DPI was employed to exclude ROS produced by NOXs. However, it must be noted that DPI is a nonspecific inhibitor of NOX. In future experiments, we will adopt a specific inhibitor of NOX or a special isoform knockout model to clarify the precise mechanisms underlying the antioxidative stress activity of GXT. Although no significant differences were evident between the GXT + MI and MI groups in terms of LVEF and FS (all *P* > 0.05), we observed an increasing trend in LVEF and FS, probably attributable to the short-term treatment and low dose of GXT. We hypothesize that longer duration and higher doses of GXT treatment should exert an obvious protective effect on cardiac function. The effects of long-term GXT treatment on ischemic myocardium require further investigation.

In conclusion, we conclude that GXT exerts a protective effect against cardiomyocyte ischemia by virtue of its antioxidative activities. GXT alleviated the degree of ischemic cardiomyocyte injury, apoptosis, and fibrosis and partly improved the cardiac function of MI rats. Furthermore, GXT suppressed the generation of ROS in cardiomyocytes and downregulated NOX2, NOX4, and p-p38 MAPK protein expression. While the precise mechanisms involved in the protective role of GXT in myocardial injury remain to be established, our results clearly support the potential utility of GXT as an effective therapeutic agent for myocardial ischemia.

## Figures and Tables

**Table 1:** Effects of GXT on echocardiographic parameters.

Parameters	Sham	MI	GXT + MI
LVEDD (mm)	5.20 ± 0.26	7.77 ± 0.38^*∗*^	6.77 ± 0.29^#^
LVESD (mm)	3.00 ± 0.26	6.30 ± 0.26^*∗∗*^	5.33 ± 0.38^#^
EF (%)	78.33 ± 7.02	43.33 ± 3.06^*∗*^	47.33 ± 8.08^*∗*^
FS (%)	43.67 ± 3.79	19.67 ± 2.52^*∗*^	25.00 ± 4.36^*∗∗*^

Data represent mean ± SD (*n* = 3). EF, ejection fraction; FS, fractional shortening; LVEDD, left ventricular end-diastolic diameter; LVESD, left ventricular end-systolic diameter; ^*∗*^*P* < 0.01 and ^*∗∗*^*P* < 0.001 versus the sham group; ^#^*P* < 0.05 versus the MI group.
